# On the improvement of heart rate prediction using the combination of singular spectrum analysis and copula-based analysis approach

**DOI:** 10.7717/peerj.14601

**Published:** 2022-12-19

**Authors:** Asieh Namazi

**Affiliations:** Department of Physical Education and Sport Science, Iran University of Science and Technology Tehran, Tehran, Iran

**Keywords:** Heart rate, Machine learning, Wearable sensors, Artificial intelligence, Prediction

## Abstract

In recent years, many people have been working from home due to the exceptional circumstances concerning the coronavirus disease 2019 (COVID-19) pandemic. It has also negatively influenced general health and quality of life. Therefore, physical activity has been gaining much attention in preventing the spread of Severe Acute Respiratory Syndrome Coronavirus. For planning an effective physical activity for different clients, physical activity intensity and load degree needs to be appropriately adjusted depending on the individual’s physical/health conditions. Heart rate (HR) is one of the most critical health indicators for monitoring exercise intensity and load degree because it is closely related to the heart rate. Heart rate prediction estimates the heart rate at the next moment based on now and other influencing factors. Therefore, an accurate short-term HR prediction technique can deliver efficient early warning for human health and decrease the happening of harmful events. The work described in this article aims to introduce a novel hybrid approach to model and predict the heart rate dynamics for different exercises. The results indicate that the combination of singular spectrum analysis (SSA) and the Clayton Copula model can accurately predict HR for the short term.

## Introduction

In recent years, many people have been working from home due to the exceptional circumstances concerning the coronavirus disease 2019 (COVID-19) pandemic. It has negatively influenced general health and quality of life. Physical inactivity imposes a significant economic burden on the health care system, and the COVID-19 pandemic has harmed physical activity (Puccinelli et al., 2021). Those who reduced their physical activity level had the highest mood disorders. Nowadays, physical activity has been gaining much attention in preventing the spread of Severe Acute Respiratory Syndrome Coronavirus. Over the last several decades, data obtained from the linear relationship between heart rate and oxygen consumption are taken to estimate maximal oxygen uptake ( ${V_O}2max$) (Jlstrand & Ryhming, 1954). For planning an effective physical activity for different clients, physical activity intensity and load degree needs to be appropriately adjusted depending on the individual physical/health conditions (Böning, Gönen & Maassen, 1984; Hoogendoorn et al., 1999).

Heart rate (HR) is one of the most critical health indicators for monitoring the physical load degree because it is closely related to the heart rate (Dong, 2016). Therefore, studying heart rate and its influencing factors is of great importance. The human body does not adapt to activities immediately, and the response heart rate is delayed and increases after a certain time of physical activity (Coats et al., 1992). Heart rate prediction estimates the heart rate at the next moment based on now and other influencing factors. Therefore, an accurate short-term HR prediction technique can deliver efficient early warning for human health and decrease the happening of harmful events or premature death (Malik & Camm, 1990; Janssen, 2012,). However, traditional heart rate prediction methods cannot meet the requirements of high-precision dynamic prediction. Thus, reliable and effective heart rate prediction is significant for preventing and controlling some cardiovascular diseases (Lei, Cai & Hua, 2021). In the last decade, various heart rate prediction models have been examined. Some models are based on a Hammerstein model’s differential equations or variants. Cheng et al. (2008) present a nonlinear condition space model for forecasting a person’s heart rate behavior based on running speed on a treadmill. Paradiso et al. (2012) employ the same model to control the bicycle heart rate. Mohammad et al. (2011) introduced the fuzzy Takagi-Sugeno model dealing with 12 parameters and is commonly used to optimization of physical activity for older, untrained people. Other model-based systems exist for running and cycling on different training devices (Su et al., 2007; Koenig et al., 2009; Leitner et al., 2014).

The work described in this article aims to introduce a novel hybrid approach to model and predict the heart rate dynamics for different exercises. Modiri et al. (2018) introduced a hybrid approach, a combination of deterministic and stochastic methods (the combination of singular spectrum analysis (SSA) and Copula-based analysis) to predict space geodetic data for the first time. The main advantage of using the SSA+Copula approach is fully describing all relations underlying different variables. SSA is not limited to using predetermined functions such as a sine wave as a base. Instead, SSA uses data-driven basis functions to extract basic components of the data series and applies a classification method to examine the relationship between the derived elements (Broomhead & King, 1986; Vautard, Yiou & Ghil, 1992). The Copula-based analysis approach exploits linear and nonlinear dependencies between variables. It is a powerful and efficient tool for dealing with multi-dimensional data and modeling the dependency structure between parameters (Joe, 1997). This study applied the combination of SSA and Copula for the first time as a novel deterministic-stochastic tool for HR prediction using only HR past data. In this method, the deterministic part is estimated by SSA, whereas Copula is used for modeling the stochastic part. This study can support the development of automated algorithms for real-time health monitoring of athletes and preventive identification of individuals at increased risk for sudden sports-related events, as suggested by Sbrollini et al. (2019).

The remainder of this article is organized as follows. “Methodology” discusses how to describe the methodology and prediction algorithms. Next, “Data Set And Data Analysis” illustrates the data set and analysis where the input data is fully described. Then, “Results” discusses the results, and finally, the conclusion is written in “Conclusions”.

## Methodology

In this study, the integration of Copula-based analysis and SSA is developed for precisely predicting heart rate.

### Singular spectrum analysis

SSA is a non-parametric technique used for many tasks, such as trend detection and extraction, de-noising, forecasting, and change-point detection. SSA works with arbitrary statistical processes, whether linear or nonlinear, stationary or non-stationary, Gaussian or non-Gaussian (Ghil et al., 2002; Hassani & Thomakos, 2010; Modiri et al., 2018).

The SSA trend extraction method can be described as having four stages (Hoseini et al., 2020; Hoseini, 2022):

- **Stage 1: Embedding.** A sliding window with length L forms a trajectory matrix **X** over the time series ${f_i}$ described as follows:

(1) $$\matrix{ {\bf X} \hfill & { = ({x_{ij}})_{i,j = 1}^{L,K} = } \hfill & {\left[ {\matrix{ {{f_1}} & {{f_2}} & {{f_3}} & \ldots & {{f_K}} \cr {{f_2}} & {{f_3}} & {{f_4}} & \ldots & {{f_{K + 1}}} \cr {{f_3}} & {{f_4}} & {{f_5}} & \ldots & {{f_{K + 2}}} \cr \vdots & \vdots & \vdots & \ddots & \vdots \cr {{f_L}} & {{f_{L + 1}}} & {{f_{L + 2}}} & \ldots & {{f_N}} \cr } } \right]\;,\;\left\{ {\matrix{ {1 < L < K} \hfill \cr {K = N - L + 1} \hfill \cr } } \right.} \hfill \cr }$$

where, **X** is a symmetric matrix with identical elements on the anti-diagonals (Korobeynikov, 2009). Also, the K represents the indices of the L-dimensional time-lagged vectors.

- **Stage 2: Decomposition.** The singular value decomposition (SVD) is applied and decomposed into the trajectory matrix **X** (Wall, Rechtsteiner & Rocha, 2003; Modiri, 2022), as ${\bf X} = {\bf U}\Sigma {V^T}$. The matrices **U** and **V** are singular and orthonormal vectors. Also, $\Sigma$ denotes a diagonal matrix of the singular values. Then, the SVD of the **X** matrix can be obtained from eigenvalues and eigenvectors of the lag-covariance matrix defined as $S = X{X^T}$ of size $L \times L$. S is named empirical orthogonal functions (EOFs) and contains eigenvalues denoted by ${\lambda _1} \ge {\lambda _2} \ge \ldots \ge {\lambda _L} \ge 0$. Then, the corresponding orthonormal eigenvectors of S can be indicated by ${U_1},{U_2}, \ldots {U_L}$ and ${V_1},{V_2}, \ldots {V_L}$ for left and right, respectively. Thus, the SVD obtained a representation $X = {X_1} + {X_2} + \ldots + {X_d}$, where the trajectory matrix can be written as: ${X_i} = \sqrt {{\lambda _i}} {U_i}V_i^T$ ( $i = 1, \ldots ,d)$. The $\sqrt {{\lambda _i}}$ is the corresponding singular value of ${{\bf X}_i}$ and d is the number of non-zero eigenvalues of ${\lambda _i}$. Each of the matrices ${{\bf X}_i}$ describes some components of the time series, and summing them should deliver ${\bf X}$.

- **Stage 3: Grouping.** The indices are split into two groups, trend and residual, as follows:

(2) $$\left\{ {\matrix{ {{X_{trend}} = {X_1} + {X_2} + \ldots + {X_I} = (\hat{x}_{i,j})_{i,j = 1}^{L,K}} \cr {{X_{residual}} = {X_{I + 1}} + {X_{I + 2}} + \ldots + {X_d}} \cr } } \right.$$

where $I \le d$.

- **Stage 4: Reconstruction.** To reconstruct the trend, averaging the anti-diagonal entries of the matrix ${{\bf X}_{trend}}$ in Eq. (2) should be done. Let *L* < *K*, then the trend of time series $G = ({g_1},{g_2}, \ldots ,{g_N})$ is:

(3) $${g_i} = \left\{ {\matrix{ {\displaystyle{1 \over i}\Sigma _{m = l}^i{{\hat X}_{m,i - m + 1}}{\rm }1 \le i \le L} \hfill \cr {\displaystyle{1 \over L}\Sigma _{m = l}^L{{\hat X}_{m,i - m + 1}}L \le i \le K} \hfill \cr {\displaystyle{1 \over {N - i + 1}}\Sigma _{m = i - K + 1}^{N - K + 1}{{\hat X}_{m,i - m + 1}}K \le i \le N} \hfill \cr } } \right.$$

where ${\hat x_{i,j}}$ is an estimation of the element ${f_{i + j - 1}}$ of the original time series.

Furthermore, the reconstructed trajectory matrix might not keep the Hankel condition, and thus ${\hat X_{i,j}} \ne {\hat X_{j,i}}$ (Li, Liu & Razavilar, 1997).

### Copula-based analysis

The Copula-based analysis exploit the dependency structure between random variables independent of their marginal distributions. It is a flexible tool that greatly improves the capture of the whole correlation pattern (Embrechts, McNeil & Straumann, 2002). The Copula-based analysis model is used for various types of studies, *e.g*., economics, hydrology, meteorology, and geodesy (Patton, 2006, 2009; Bárdossy & Li, 2008; Bárdossy & Pegram, 2009; Laux et al., 2011; Modiri, 2015; Modiri et al., 2018, 2020).

A Copula model is revealed as a function C from ${[0,1]^2}$ to [0, 1]. The general properties of Copula can be itemized as follows (Genest & Rivest, 1993; Nelsen, 2007):

-
$C(u,0) = C(0,v)$,
-
$C(u,1) = u$ and $C(1,v) = v$,
- The Copula is unique when the marginals are continuous functions.

Besides, it should be mentioned that the variables transformed by any monotonic increasing functions will not affect its Copula. The empirical Copula is an estimator for the unknown theoretical Copula and is defined on the rank space as (Genest & Favre, 2007):

(4) $${C_e}(u,v) = \displaystyle{1 \over n}\sum\limits_{i = 1}^n {\bf 1} \left (\displaystyle{{{r_i}} \over {n + 1}} \le u,\displaystyle{{{s_i}} \over {n + 1}} \le v \right)$$

where, $({r_1}), \ldots ,({r_n})$ mean the pairs of ranks of the variable $({x_1}),({x_2}), \ldots ,({x_n})$. $({s_1}), \ldots ,({s_n})$ denote the pairs of ranks of the variable $({y_1}),({y_2}), \ldots ,({y_n})$. $n$ is the length of the data vector. A total of **1** is the indicator function. The most commonly used Copula model is the Archimedean Copula model, which demonstrates a-/symmetrical upper/lower tail dependency and can be estimated directly in a simple form.

(5) $$C(u,v) = {\phi ^{ - 1}}\{ \phi (u) + \phi (v),\theta \}$$

where $\theta$ is the Copula parameter and the function $\phi$ is the Copula generator with the following characteristics (Nelsen, 2007).

- **Clayton Copula.** The Clayton Copula is an asymmetric Archimedean Copula, which can be used for the variables with a lower tail dependence structure. In order to define the Clayton Copula model, first, its generator should be estimated as follow (Clayton, 1978):

(6) $${\phi ^{Cl}}(x) = \displaystyle{1 \over \theta }({t^{ - \theta }} - 1)$$

where, $\theta$ is the Clayton Copula parameter given over $- 1 \le \theta$. Therefore, the Clayton Copula can be simplified to Nelsen (2007):

(7) $${C_\theta }(u,v) = \max {[({u^{ - \theta }} + {v^{ - \theta }} - 1),0]^{ - \displaystyle{1 \over \theta }}}$$

- **Frank Copula.** The Frank Copula is mainly used to model the symmetrical dependency between the variables. So, the generator of the Frank Copula model is indicated by Nelsen (2007):

(8) $${\phi ^{Fr}}(t) = - \ln \left \{ \displaystyle{{{e^{ - \theta t}} - 1} \over {{e^{ - \theta }} - 1}}\right\}$$

The Frank Copula parameter is defined over $- \infty < \theta < \infty$ and its formula is given by Nelsen (2007):

(9) $${C_\theta }(u,v) = \displaystyle{1 \over \theta }\ln (1 + \displaystyle{{({e^{ - \theta u}} - 1)({e^{ - \theta v}})} \over {{e^{ - \theta }} - 1}})$$

In the case of independence Copula, the Frank Copula parameter is equal to zero, and the higher parameter shows a linear dependency structure between variables.

- **Gumbel Copula.** The Gumbel Copula is also an asymmetric Archimedean Copula, which is used to capture the upper tail dependence. The Gumbel Copula generator is shown as follows:

(10) $${\phi ^{Gu}}(t) = {( - \ln t)^\theta }$$

The Gumbel Copula parameter is defined over $1 \le \theta$ and its formula is given by Nelsen (2007):

(11) $${C_\theta }(u,v) = {e^{ - {{(( - \ln {{(u)}^\theta }) + ( - \ln {{(v)}^\theta }))}^{\textstyle{1 \over \theta }}}}}$$

In the case of independence structure between the variables, the Gumbel Copula parameter is equal to 1 and if the $\theta \to \infty$ is the comonotonicity Copula. All described Copula models are obtained by estimating the Copula parameters and, in this study, are derived from the maximum likelihood (ML) estimation described by Joe (1997).

### Prediction algorithm

The Copula + SSA is developed as a novel hybrid method to predict different parameters. It uses training data to model the data’s underlying structure.

**Figure 1:**
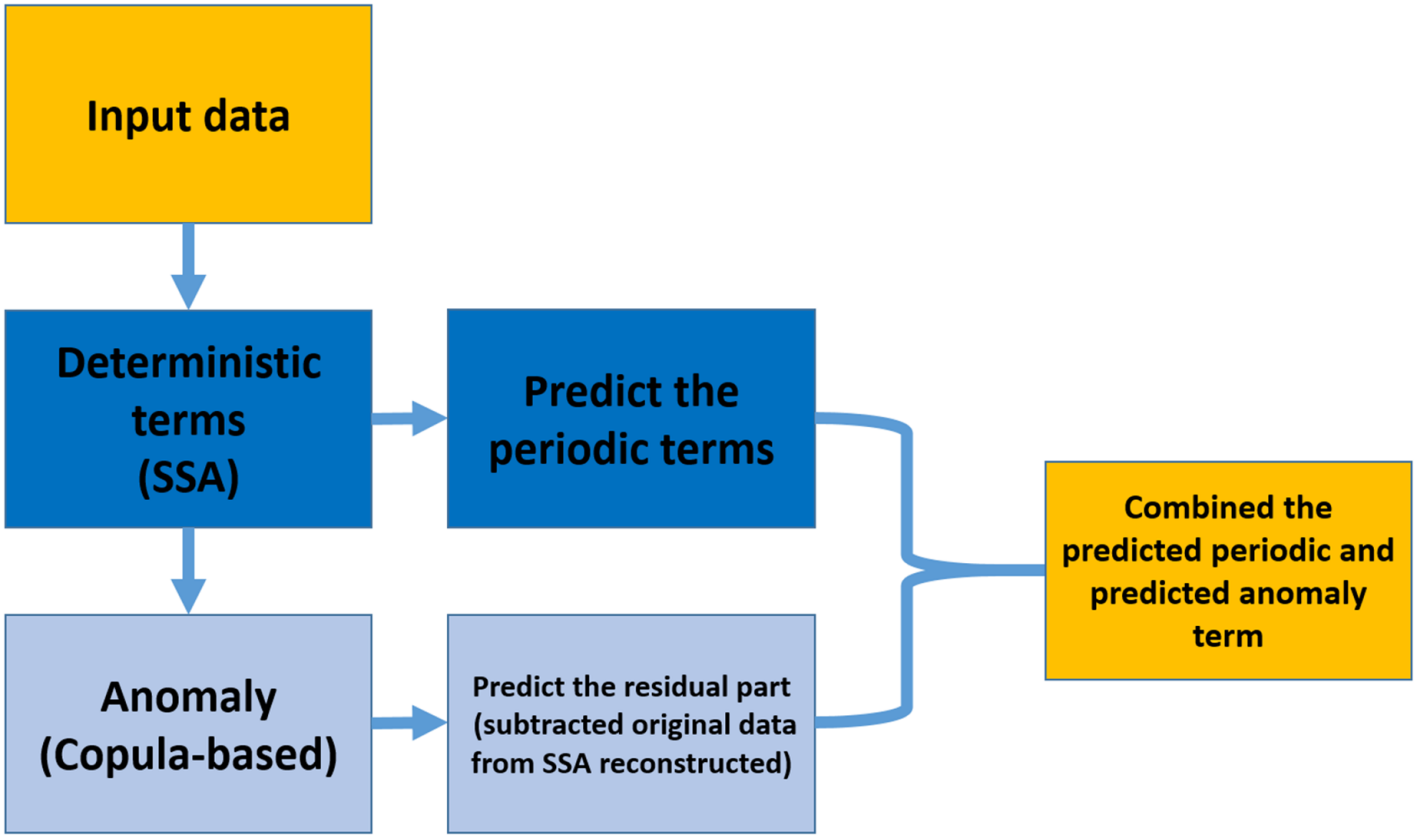
Copula + SSA algorithm for modeling and predicting the data by using the information within the past data.

As shown in Fig. 1, the prediction algorithm consists of two parts; SSA models the deterministic part, and the anomaly (the residual of subtracted data by the reconstructed time series) part is modeled by Coupla-based analysis. Then, the SSA extrapolates the deterministic component, and the residual part is predicted by Copula-based analysis. In the end, the SSA extrapolated trend and predicted anomaly would be combined (Modiri, 2021).

## Data set and data analysis

In this article, the HR data are from the online available data set: ‘Sport Database: Cardiorespiratory data acquired through wearable sensors while practicing sports’ (Sbrollini et al., 2019). All subjects gave their informed consent prior to data collection and acquisitions, which were undertaken in compliance with the ethical principles of Helsinki Declaration and approved by the institutional expert committee. The characteristics of the HR signal is shown in Table 1.

**Table 1:** Characteristics of the HR signals.

Signal	Sampling frequency	Amplitude range
Heart rate	1 Hz	25–240 bpm

**Figure 2:**
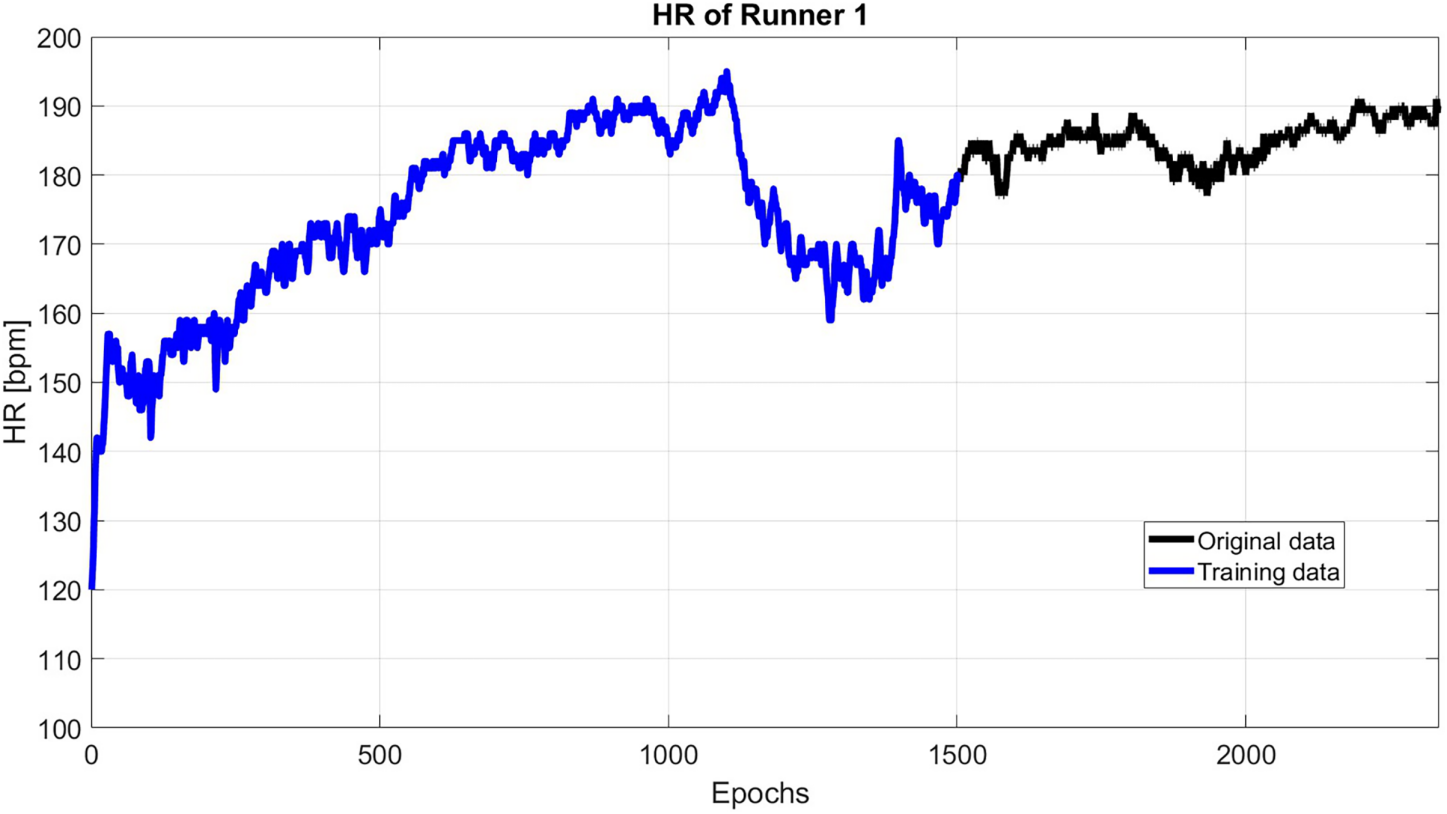
The original HR data (black) and the reconstructed HR data (blue).

In this study, the data from ten subjects who did running test is used. A particular survey protocol was characterized for running. It contains a 6.1 Km route around the city of Ancona, Italy (see Fig. 2 of the reference Sbrollini et al. (2019)). The protocol contains four phases with different slopes:

- Initial flat phase (1.3 km long with a 0)
- Uphill phase (1.2 km long with a $6.8\%$ slope).
- Downhill phase (1 km long with a $7.2\%$ slope).
- Final flat phase (2.6 km long with a $0\%$ slope)

All subjects were supposed to be healthy (*i.e*., no previous history of cardiorespiratory diseases and not taking any drug) at the acquisition time. Also, the demographic data of the Sport Database is given by Table 2. All subjects gave their informed consent prior to data collection and acquisitions, which were undertaken in compliance with the ethical principles of the Helsinki Declaration and approved by the institutional expert committee.

**Table 2:** Demographic data of sport database.

Number of subjects	Number of CRD	Gender M/F	Age (years)	Weight (kg)	Height (cm)	Smoking NO/YES	Alcohol consumption NO/SOMETIMES	Weekly training rate
10	10	9/1	22 ± 3	70 ± 6	179 ± 7	5/5	1/9	3 ± 1

As an example, Fig. 2 shows the HR of subject 1, where this subject has an initial flat phase: from 00:00:00 to 00:07:45, then has an uphill phase: from 00:07:45 to 00:18:32, after that, the downhill phase: from 00:18:32 to 00:25:47, in the end, the subject starts the final flat phase: from 00:25:47 to 00:43:54.

The measured HR data could be divided into two parts. The first part deals with the trend and deterministic term of HR. So, SSA is employed to model the HR signal’s main behavior (trend + deterministic term). Then, the difference between the HR and SSA estimated data is modeled using the Copula-based analysis approach. After that, the main behavior of HR is extrapolated using the SSA *a priori* model. Correspondingly, the anomaly part is predicted using the Copula-based model. Finally, the anomaly solution is added to the SSA-forecasted data.

Therefore, the analysis of the data is divided into two main steps.

### SSA trend estimation and extrapolation

As it shown in Fig. 2, the first 1,500 epochs are taken as training data with sliding step 1. Then, the first 60 singular value is used to reconstruct the HR. Then, the reconstructed HP is extrapolated up to the next 30 s.

### Copula anomaly modeling and prediction

**Figure 3:**
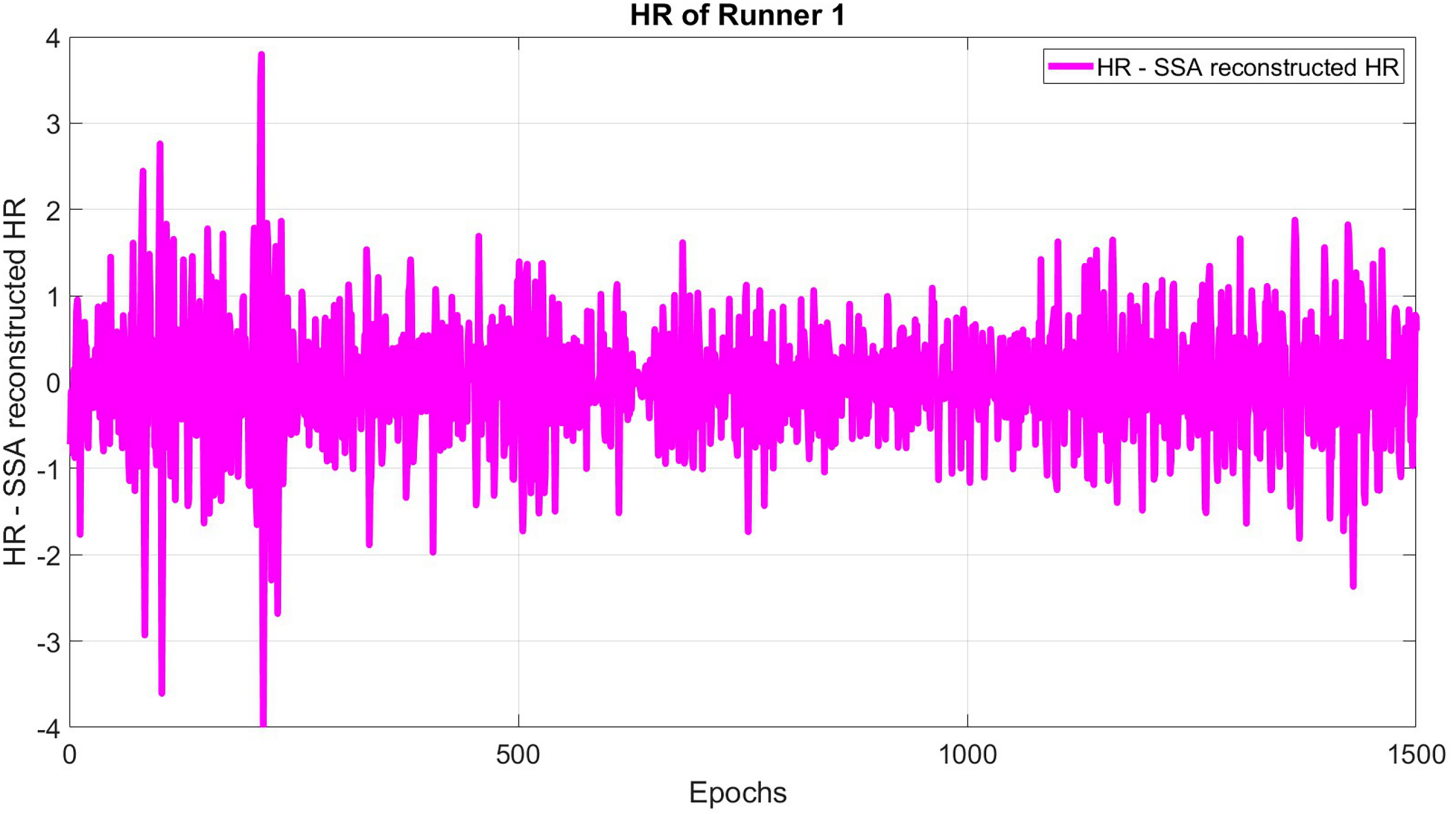
The difference between original and reconstructed time series for HR.

The subtracted original HR and SSA reconstructed HR is formed using a window length of 30 and epoch delay of 1 epoch (see Fig. 3):

- The marginal distribution of each column of the matrix is computed.
- It is transformed into the rank space.
- The empirical Copula between column i and i + 1 is computed.
- Eventually, the Clayton, Frank, and Gumbel Copula are fitted to empirical Copula by applying the appropriate goodness of fit test. After that, 3,000 random data are sampled from the conditional Copula CDF and transformed back to the data space using inverse marginal. Thus, the mean value of 3,000 random data is computed.

**Figure 4:**
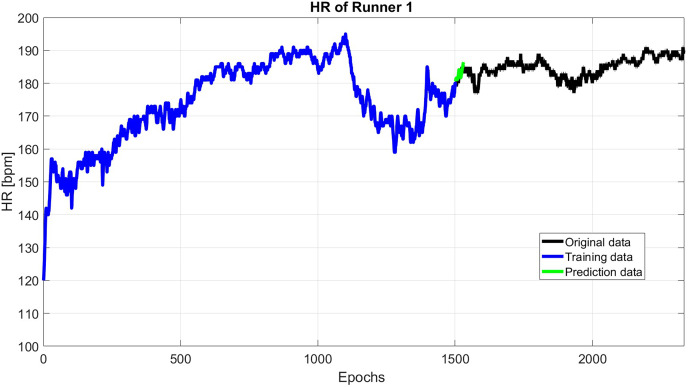
A total of 30 s HR prediction using 1,500 past epochs.

Finally, the HR predicted data is the sum of the results of predicted periodic terms using SSA and predicted anomaly using the Copula-based model (see Fig. 4).

## Results

**Figure 5:**
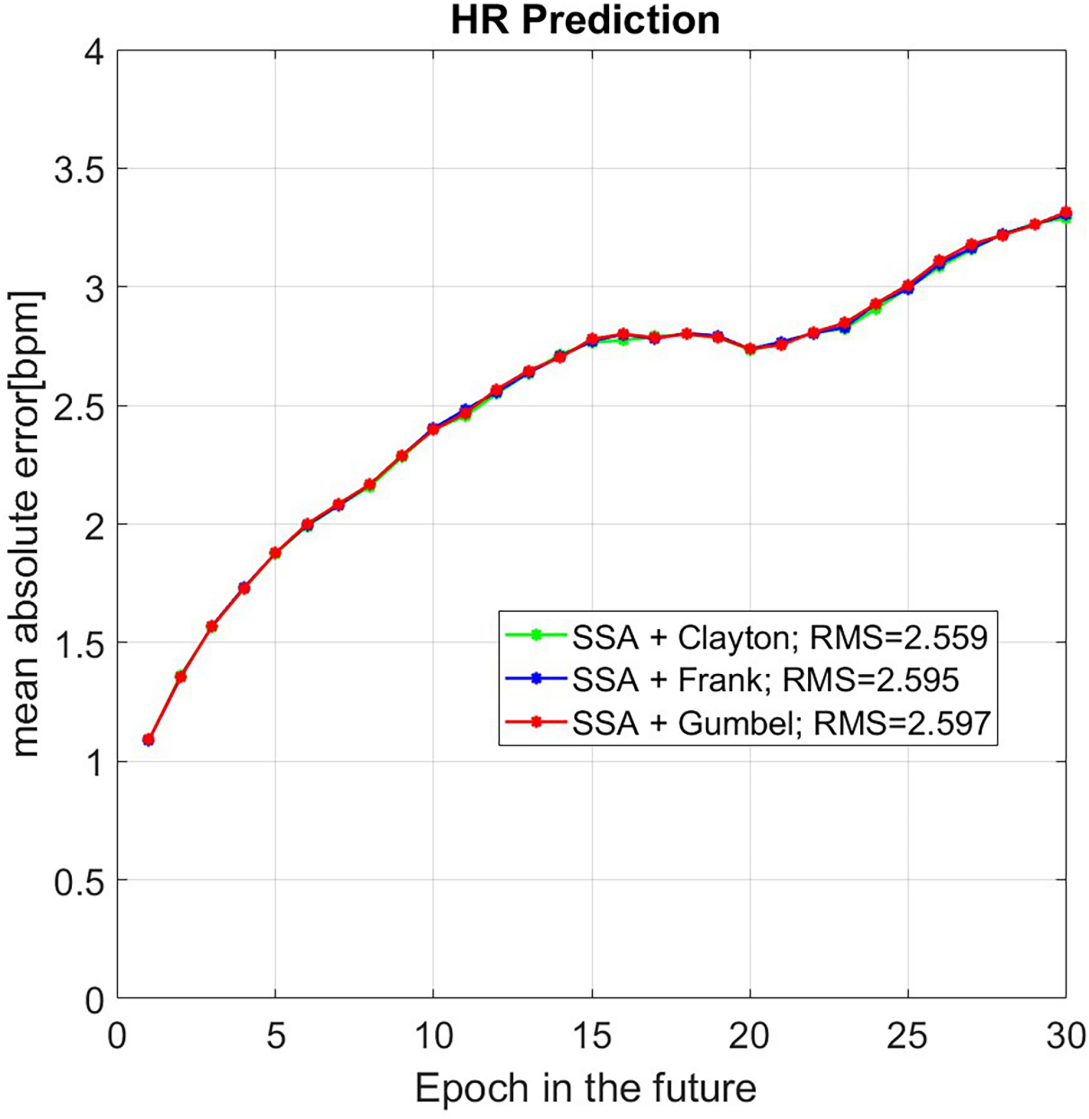
Mean value of MAE of HR prediction for 508 data set with the unit is [bpm].

We utilized 1,500 epochs of HR data for the 30-epochs-ahead prediction. To verify the reliability of this method, the results were compared with the original data. As a result, almost 502 sets of predicted HR have been done. So, Fig. 5 shows the mean value of MAE for different prediction algorithms (SSA+Clayton (green), SSA+Frank (blue), SSA+Clayton (red)).

In Fig. 5, the results of SSA+Copula are displayed in green, blue, and red for Clayton, Frank, and Gumbel Copula, respectively. The result of SSA + Clayton Copula shows slightly better performance.

The results demonstrated in this study, SSA + Copula technique performed better compared to the techniques shown in the recent studies by Alharbi et al. (2021), Monkaresi, Calvo & Yan (2013), Oyeleye et al. (2022). The mean of MAE is lower than 2 bpm up to the first seven epochs and slightly increases up to 3.3 bpm for the subsequent 30 epochs. Among the proposed techniques, SSA + Clayton Copula method performs better than SSA + Frank and SSA + Gumbel Copula.

**Figure 6:**
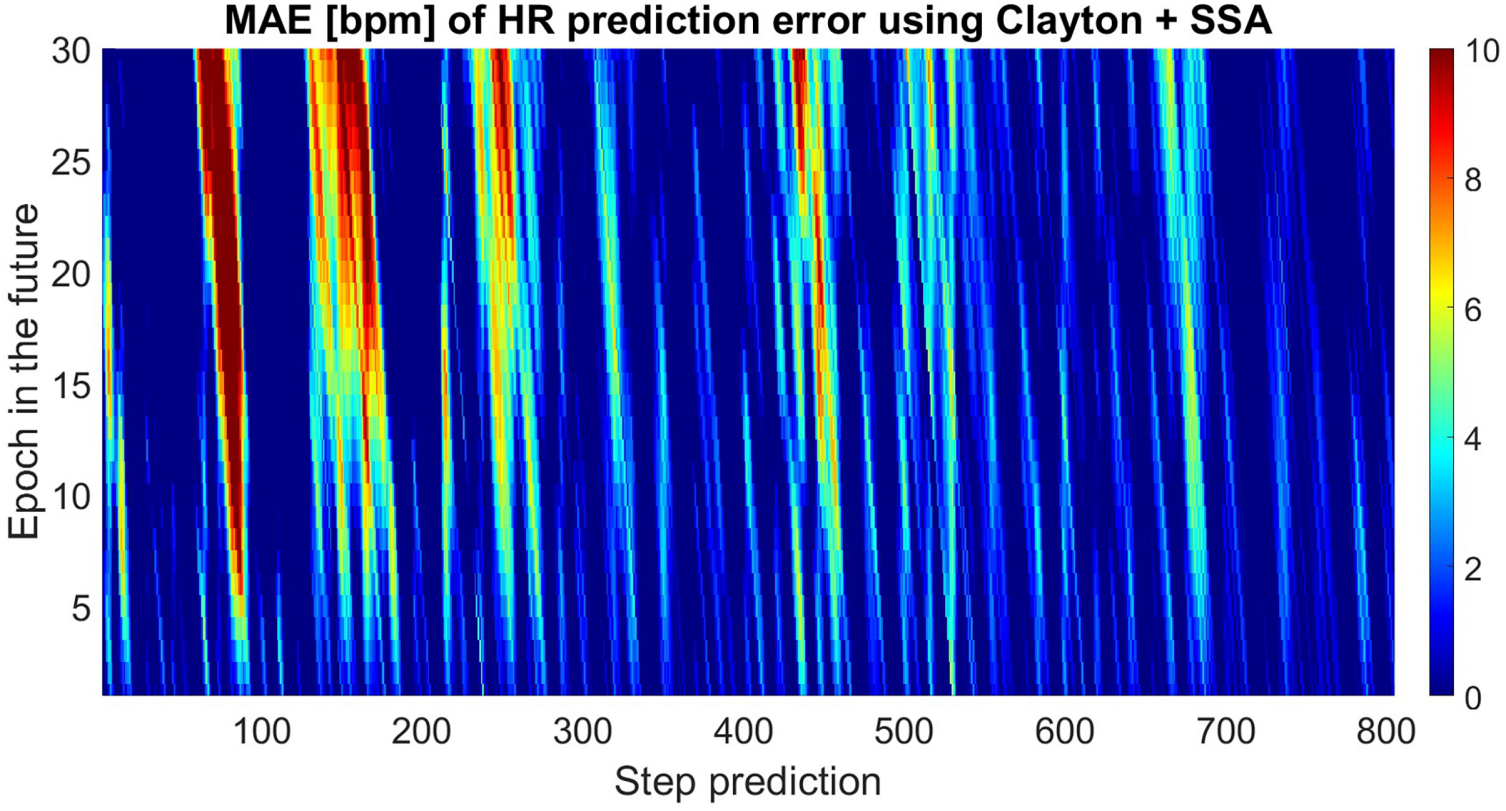
Absolute errors of the predicted HR using SSA+Clayton Copula compared with original HRdata. The unit is [bpm].

Figure 6 presents the absolute error of 30-epochs-ahead prediction for almost 805 predicted data sets. The proposed method did not always perform better, and the results were not as good as we expected. This may have been caused by changes in the amplitudes of the deterministic terms where the SSA could not capture all features. In order to predict more precisely, we would have to increase the interval of training time.

## Conclusions

This study proposed a new prediction technique to predict HR, one of the most important health indicators for planning physical activity. Then, novel prediction techniques have been used to detect early warnings for human health. The best-developed model for each case with the lowest MAE is used to evaluate the prediction methods in real-time. The results illustrate that the proposed method could efficiently and precisely predict HR. As demonstrated, the Copula-based analysis successfully increases the accuracy of HR prediction by modeling the stochastic part of the HR. However, as described in Modiri et al. (2018), the primary error contributions come from the SSA extrapolation part. So, further investigations about the SSA training time will be required to clarify this issue. Also, the Copula-based analysis can potentially add more physical parameters for HR prediction. Therefore, future investigations are needed to get benefit from other parameters.

## Additional Information and Declarations

### Data Availability

The following information was supplied regarding data availability: The raw data is available at Agnese Sbrollini, Micaela Morettini, Elvira Maranesi, Ilaria Marcantoni, Amnah Nasim, Roberta Bevilacqua, Giovanni R. Riccardi, Laura Burattini. 2019. Sport Database: Cardiorespiratory data acquired through wearable sensors while practicing sports. 27:104793, https://doi.org/10.1016/j.dib.2019.104793.
